# Evolution of the MLO gene families in octoploid strawberry (*Fragaria* ×*ananassa*) and progenitor diploid species identified potential genes for strawberry powdery mildew resistance

**DOI:** 10.1038/s41438-021-00587-y

**Published:** 2021-07-01

**Authors:** Ronald R. Tapia, Christopher R. Barbey, Saket Chandra, Kevin M. Folta, Vance M. Whitaker, Seonghee Lee

**Affiliations:** 1 Department of Horticultural Sciences, University of Florida, IFAS Gulf Coast Research and Education Center, Wimauma, FL 33598 USA; 2 Department of Horticultural Sciences, University of Florida, 1301 Fifield Hall, PO Box 110690, Gainesville, FL 32611 USA

**Keywords:** Plant breeding, Genomics

## Abstract

Powdery mildew (PM) caused by *Podosphaera aphanis* is a major fungal disease of cultivated strawberry. *Mildew Resistance Locus O* (*MLO*) is a gene family described for having conserved seven-transmembrane domains. Induced loss-of-function in specific *MLO* genes can confer durable and broad resistance against PM pathogens. However, the genomic structure and potential role of *MLO* genes for PM resistance have not been characterized yet in the octoploid cultivated strawberry. In the present study, *MLO* gene families were characterized in four diploid progenitor species (*Fragaria vesca*, *F*. *iinumae*, *F*. *viridis*, and *F*. *nipponica*) and octoploid cultivated (*Fragaria ×ananassa)* strawberry, and potential sources of *MLO*-mediated susceptibility were identified. Twenty *MLO* sequences were identified in *F. vesca* and 68 identified in *F. ×ananassa*. Phylogenetic analysis divided diploid and octoploid strawberry *MLO* genes into eight different clades, in which three *FveMLO* (*MLO10*, *MLO17*, and *MLO20*) and their twelve orthologs of *FaMLO* were grouped together with functionally characterized *MLO* genes conferring PM susceptibility. Copy number variations revealed differences in *MLO* composition among homoeologous chromosomes, supporting the distinct origin of each subgenome during the evolution of octoploid strawberry. Dissecting genomic sequence and structural variations in candidate *FaMLO* genes revealed their potential role associated with genetic controls and functionality in strawberry against PM pathogen. Furthermore, the gene expression profiling and RNAi silencing of putative *FaMLO* genes in response to the pathogen indicate the function in PM resistance. These results are a critical first step in understanding the function of strawberry *MLO* genes and will facilitate further genetic studies of PM resistance in cultivated strawberry.

## Introduction

The *Mildew Resistance Locus O* (*MLO*) gene family is present in several crop species and was described for having conserved seven-transmembrane (TM) and C-terminal calmodulin-binding (CaMB) domains that are functionally important for powdery mildew resistance^1,2^. The genetic diversity among *MLO* gene members has been explored in a wide variety of plant species^3–5^, but still little information is known about its origin and biochemical function associated with plant development and response to biotic and abiotic stresses. A recent study revisited the biological origin of *MLO* gene family that traced it back from the ancestral species of unicellular photosynthetic eukaryote and, that in the course of evolution diversified to the present MLO proteins^6^. In modern plants, the divergence of MLO proteins was demonstrated through phylogenetic analysis and classified them to different clades ranging from four to eight^5,7–9^.

The *MLO* gene family has been the focus of attention in many crop species because transgenic downregulation or elimination of specific endogenous *MLO* genes has led to powdery mildew (PM) resistance^10^. However, alteration of *MLO* gene sequences can trigger negative phenotypic effects including premature leaf chlorosis, altered root growth and pollen tube germination^11^. The first *MLO*-based resistance trait was first characterized in barley (*HvMLO*), where a loss-of-function mutation in an *MLO* gene conferred broad resistance against PM pathogens^12^. This discovery led to subsequent comparative genomic studies of *MLO* gene families in several plant species to find suitable candidate genes. In the model plant *Arabidopsis thaliana*, three *MLO* genes (*AtMLO2*, *AtMLO6*, and *AtMLO12*) were functionally characterized as conferring PM susceptibility^13^. Expression analysis of these homologs upon pathogen challenge suggested functional redundancy. The *Atmlo2* single mutant has only partial resistance while triple mutants (*Atmlo2, Atmlo6*, and *Atmlo12*) have full resistance against PM pathogens^7^. Since identification of functional *MLO* genes is enabled by DNA sequence information, the advent of reference genomes for several crop species provides an opportunity to identify *MLO* orthologs as targets for functional studies. *MLO* genes have been genetically characterized across many crops including apple (*MdMLO11* and *MdMLO19*)^5^, pepper (*CaMLO2*)^14^, rose (*RhMLO1)*^15^, grapevine (*VvMLO6* and *VvMLO7*)^16^, melon (*CmMLO2*)^17^, cucumber (CsMLO1)^18^, pea (*PsMLO1*)^19^, tobacco (*NtMLO1*)^20^, tomato (*SlMLO1*)^21^, rice (*OsMLO2*)^22^, corn (*ZmMLO1*)^23^, and wheat (*TaMLO1*)^22,24^. Recently, targeted-genome mutation of *SlMLO1* resulted in the development of a PM-resistant *Slmlo1* tomato variety^25^. The potential functional role of these genes as susceptibility factors has not been highlighted yet in the octoploid cultivated strawberry. Thus, in the present study, the orthologs of *AtMLO2*, *AtMLO6* and *AtMLO12* were identified from the octoploid strawberry genome and explored their possible function for PM resistance.

Strawberry PM caused by an obligate parasite *Podosphaera aphanis* (Wallr.) (former *Sphaerotheca macularis* f. sp. *fragariae*)^26^ is a major fungal plant disease in strawberry. PM primarily affects the leaf and, depending on severity, can also affect other organs^27^. Initially, a white powdery mycelium develops on the underside of the leaves, followed by upward curling of the leaf edges, while severe leaf infection can cause burning at the leaf margin^28^. Infected flowers and fruits may result in fruit deformation and delayed ripening^29^. PM is widespread in many strawberry growing regions worldwide, such that both open-field and high-tunnel growing systems may experience severe yield losses when infected fields are left untreated^30^. Most farmers rely on multiple pesticide applications to manage PM. Hence, developing cultivars with improved resistance is highly advantageous.

The modern cultivated strawberry (*Fragaria ×ananassa*) is an allo-octoploid (2*n* = 8*x* = 56) resulting from hybridization between a Chilean strawberry (*F. chiloensis*) and a North American native strawberry (*F. virginiana*)^31^. Further domestication of *F. ×ananassa* produced large and flavorful berries that have become the world’s most widely grown fruit crop. In 2010, the genome of the diploid progenitor species *F. vesca* was sequenced^32^ and has been used as a diploid reference genome towards molecular marker development^33^ and for gene-trait association studies in *F. ×ananassa*^34,35^. Recently, three other diploid reference sequences, *F*. *iinumae*^36^, *F*. *nipponica*^37^, and *F*. *viridis*^38^, have been published (available at https://www.rosaceae.org/). However, the octoploid *F. ×ananassa* genome is far more complicated than its diploid progenitor. In 2019, the chromosome-scale *F. ×ananassa* ‘Camarosa’ reference genome was developed, and *F. vesca* was shown to be the dominant diploid progenitor in terms of gene content, expression abundance and genetic control for metabolic and disease-resistance traits^39^. This new reference sequence will serve as a powerful genetic resource to unravel the complexity of octoploid strawberry genome for gene-trait association studies, including causal *MLO* genes in strawberry breeding programs.

The goals of this study were to characterize the *MLO* gene family in four diploid progenitors (*F. vesca*, *F*. *iinumae*, *F*. *viridis*, and *F*. *nipponica*) and octoploid (*F. ×ananassa*) strawberry using currently available high-quality genome sequences. The genomic structures of *MLO* genes were compared between diploid and octoploid strawberry, and patterns of transcript levels were explored throughout different strawberry plant tissues. Furthermore, we determined potential *MLO* genes associated with PM resistance in cultivated strawberry. These data and insights generated here are a critical first step in understanding the function of *MLO* genes in strawberry and will facilitate further genetic studies of PM resistance in cultivated strawberry.

## Results

### Genome-wide identification of the *MLO* gene family in diploid and octoploid strawberry

**Fig. 1:**
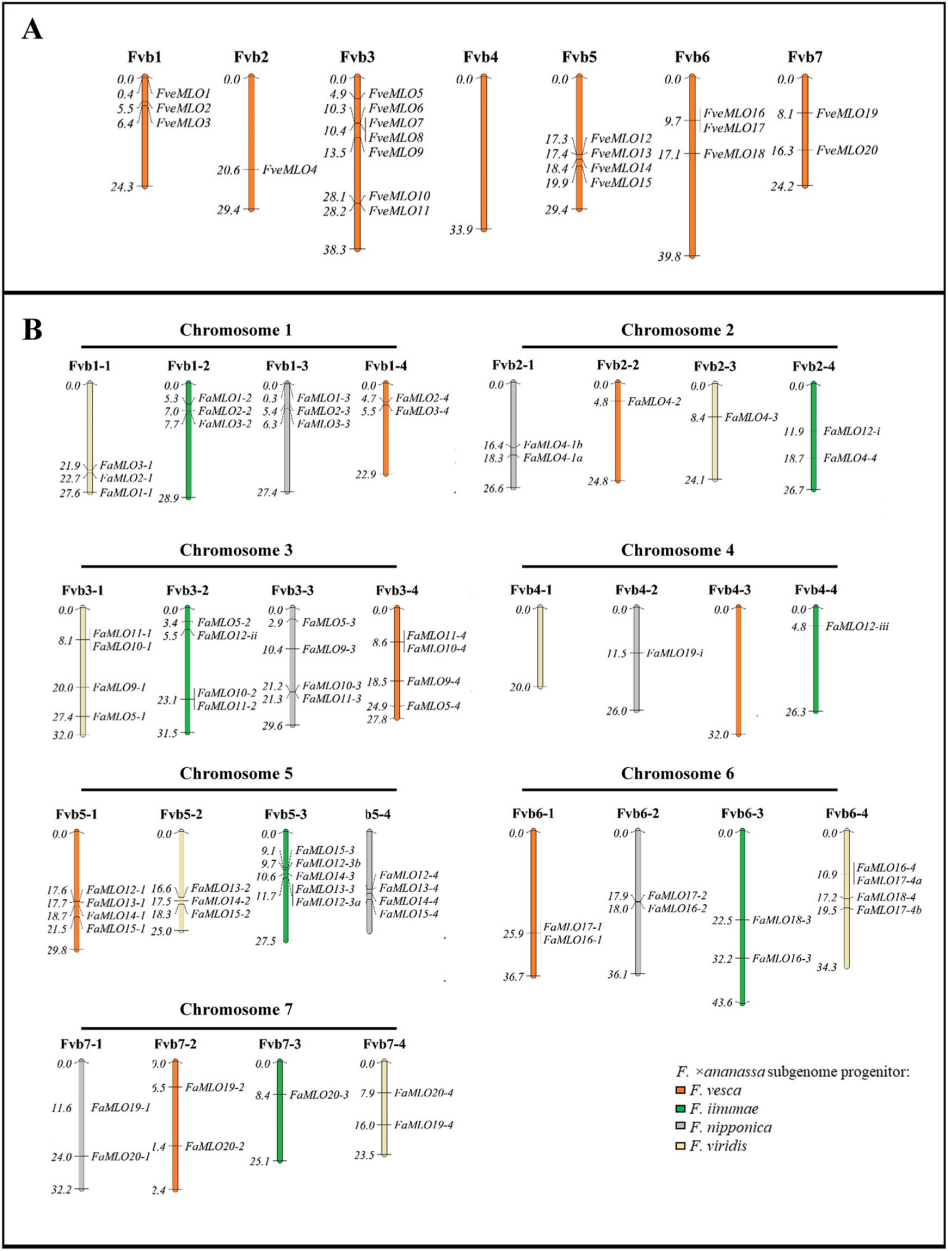
Chromosomal localization and distribution of *FveMLO*. **A** and *FaMLO* **B** genes in *F. vesca* and *F. ×ananassa* genomes, respectively. The seven chromosomes of *F. vesca* were named as Fvb1–Fvb7 while *F. ×ananassa* was named as Fvb1–1 to Fvb1–4, Fvb2–1 to Fvb2–4, Fvb3–1 to Fvb3–4, Fvb4–1 to Fvb4–4, Fvb5–1 to Fvb5–4, Fvb6–1 to Fvb6–4 and Fvb7–1 to Fvb7–4, respectively, to indicate four subgenomes within each chromosome. Different color of *F. ×ananassa* subgenomes indicates their respective diploid progenitors, namely *F. viridis* (yellow), *F. iinumae* (green), *F. nipponica* (gray), and *F. vesca* (orange)^39^ Putative strawberry *MLO* gene chromosome locations were visualized using MapChart 2.3 software26. The relative chromosome size was indicated by the unit Mbp.

Using the Arabidopsis AtMLO1 (AT4G02600) protein sequence as a BLAST query, 20 putative *MLO* genes were identified using the latest diploid genome annotation *F. vesca* v4.0.a1^40^. These 20 *MLO* genes were renamed *FveMLO1* through *FveMLO20* based on their ordered chromosomal positions (Fig. 1A, Table S1-A). Predicted proteins ranged between 200 and 903 aa with an average of 524 aa. Three truncated MLO proteins were identified as putative pseudogenes (Table S2-B). The number of predicted *MLO* genes in diploid strawberry agrees with the previous characterization of strawberry *MLO* genes using the first *F. vesca* draft genome^41^.

The *FveMLO* genes identified in *F. vesca* were then used to identify gene orthologs in the octoploid strawberry-annotated genome *F. ×ananassa* v1.0.a1(hardmasked)^39^. Analysis revealed 68 predicted *MLO* genes across 28 chromosomes (Fig. 1B, Table S1-B). Of 20 *FveMLO* genes, 17 were matched with high amino acid sequence identity to a putative ortholog; however, conserved sequences could not be found for *FveMLO6*, *FveMLO7*, or *FveMLO8*. Based on their putative orthology to *F. vesca*, the octoploid *MLO* genes were named *FaMLO1* through *FaMLO20*, respectively. To distinguish homeologous *MLO* genes, *FaMLO1–1 to FaMLO1–4* were used for each *FveMLO1* homolog. Predicted MLO proteins have amino acid sequence lengths ranging between 144 and 2,365 with an average of 560 amino acids (Table S2).

**Fig. 2:**
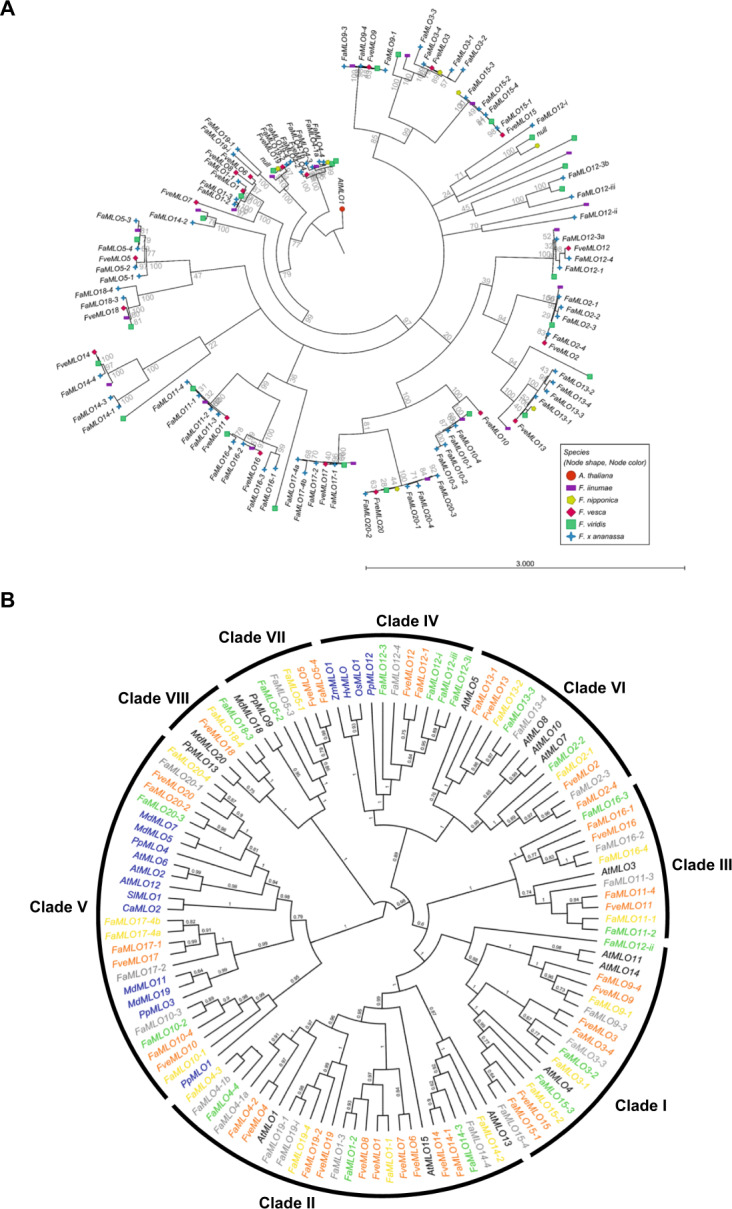
Phylogenetic analysis of *MLO* gene family in strawberry and other related crop species. **A** The maximum likelihood tree of *MLO* coding sequences from *F. ×ananassa* and four putative diploid progenitors, *F. vesca*, *F. iinumae*, *F. nipponica*, and *F. viridis*. Arabidopsis MLO1 was used as an outgroup. *Fragaria* spp. are indicated by node shapes and node color. **B** Phylogenetic tree of MLO protein sequences of strawberry species *F. vesca* and *F. ×ananassa*, and other plant species such as Arabidopsis, tomato, pepper, rice, barley, maize, apple, and peach. Strawberry *MLO* genes together with other *MLOs* were grouped into eight different clades. Unrooted phylogenetic tree of 15 *AtMLOs*, 20 *FveMLOs*, 59 *FaMLOs*, five *PpMLOs*, six *MdMLOs*, *CaMLO2*, *SlMLO1, HvMLO, OsMLO1*, and *ZmMLO1* was constructed by using FastTree via Geneious software. FaMLO proteins were highlighted in different colors according to their subgenomic location, the respective diploid progenitors such as the *F. viridis*-like (yellow), *F. vesca*-like (orange), *F. nipponica*-like (gray) and *F. iinumae*-like (green) subgenomes. MLO proteins highlighted in blue were previously characterized MLO-susceptible genes.

Twenty *FveMLO* genes were distributed randomly across six chromosomes of the diploid *F. vesca* genome: three in Fvb1, one in Fvb2, seven in Fvb3, four in Fvb5, three in Fvb6, and two in Fvb7 (Fig. 1, Table S1-A). The coding DNA sequence composition ranged from 1 to 16 exons (Fig. S1-A). In the octoploid genome, 68 *FaMLO* genes were distributed across every chromosome, except Fvb4–1 and Fvb4–3 (Fig. 1B, Table S1-B). The intron–exon structures of *FaMLO* genes have more variation as compared with the diploid progenitor *F. vesca*, with coding DNA sequence (CDS) composition ranging from 1 to 23 exons (Fig. S1-B). Despite this structural complexity, many *FaMLO* genes demonstrated high DNA sequence identity with the diploid progenitors, *F. vesca*, *F. iinumae*, and *F. nipponica* (Fig. 2 and Fig. S2). Two additional *FaMLO* sequences in chromosome 4–2 (*FaMLO19-i*) and 4–4 (*FaMLO12-iii*) were identified in *F. ×ananassa* that was not present in *F. vesca* (Fig. 1A, B).

Structural gene variation, especially between homeologous genes, could imply that some homeologs evolved differently in regulation of gene expressions and could result in novel function^42,43^. Putative strawberry *MLO* genes were selected for further phylogenetic analysis of functional divergence.

### Phylogenetic relationship analysis of *MLO* genes in diploid and octoploid strawberry

To study the evolutionary relationships of *MLO* genes in *F. ×ananassa* with its diploid progenitors, we created the maximum likelihood tree of *MLO* coding sequences from octoploid *F. ×ananassa* and diploids *F. vesca, F. iinumae, F. nipponica*, and *F*. *viridis* (Fig. 2A). This phylogeny demonstrates that a majority *FaMLO* genes, as well as their homeologs, are most similar to a *Fragaria vesca* ortholog. The overrepresentation of *F. vesca*-like *FaMLO* genes supports the recent finding of *F. vesca* subgenome dominance in octoploid strawberry, which occurred via homeologous exchange between subgenomes of octoploid strawberry and resulted in an octoploid genome that is broadly more *F.vesca-*like^39^. Strong homology with *F. iinumae* can be observed within a few octoploid *MLO* homeologous groups, including *FaMLO3, MLO16, and MLO20*, and possibly signify instances of conversion by the *F. iinumae* subgenome. Homology with the *F. nipponica MLO* sequences is rarer, likely due to the noted conversion away from this subgenome in the modern octoploid^39^ and due to the incompleteness of the *F. nipponica* genome (Fig. 2A).

To estimate the direction and magnitude of evolutionary pressures on *FaMLO* proteins, we applied a pairwise comparison of *MLO* coding sequences from octoploid and diploid strawberry spp., and calculated the rate of nonsynonymous (*d*N) and synonymous (*d*S) mutations among putative orthologs (Table S3). Most *FaMLO* genes demonstrate evidence of negative selection, with their low *d*N/*d*S ratios indicating a functional protein constraint. A few *FaMLO* homeologs, including *FaMLO16-1* and *FaMLO20-3*, appear to be under neutral selection (Table S3). No *FaMLO* genes showed evidence of positive selection (*d*N/ *d*S » 1).

To study the evolutionary relationships between strawberry *MLO* genes and functional *MLO* genes from other plant species, we aligned the deduced amino acid sequence of 20 *FveMLO*s and 68 from *FaMLOs* with previously characterized *MLOs* from Arabidopsis (*AtMLO*s), corn (*ZmMLO1*), rice (*OsMLO1*), barley (*HvMLO*), tomato (*SlMLO1*), pepper (*CaMLO2*) and other rosaceous crops such as apple (*MdMLO*s) and peach (*PpMLO*s). Phylogenetic analysis using MUSCLE and FastTree divided *MLO* genes into eight different clades (Fig. 2B). Functionally characterized *MLO* genes that are associated with PM susceptibility from selected monocots and dicots were clustered in clades IV and V, respectively^7^. Among strawberry *MLO* genes, *FveMLO12* and *FaMLO12* together with *HvMLO, OsMLO1*, and *ZmMLO1* were grouped in clade IV, while *FveMLO10*, *FveMLO17*, *FveMLO20, FaMLO10, FaMLO17*, and *FaMLO20* together with *SlMLO1, CaMLO1, AtMLO2, AtMLO6*, and *AtMLO12* were grouped in clade V. The remaining putative *MLO* sequences were distributed to six other groups. Thus, three *FveMLO* (*FveMLO10*, *FveMLO17*, and *FveMLO20*) and 12 *FaMLO* (*FaMLO10-1, FaMLO10-2, FaMLO10-3, FaMLO10-4, FaMLO17-1, FaMLO17-2, FaMLO17-4a, FaMLO17-4b, FaMLO20-1, FaMLO20-2, FaMLO20-3*, and *FaMLO20-4*) proteins have a close evolutionary relationship with the MLO proteins known to confer PM susceptibility in diploid plants (Fig. 2B).

### Synteny analysis of octoploid strawberry *MLO* genes

**Fig. 3:**
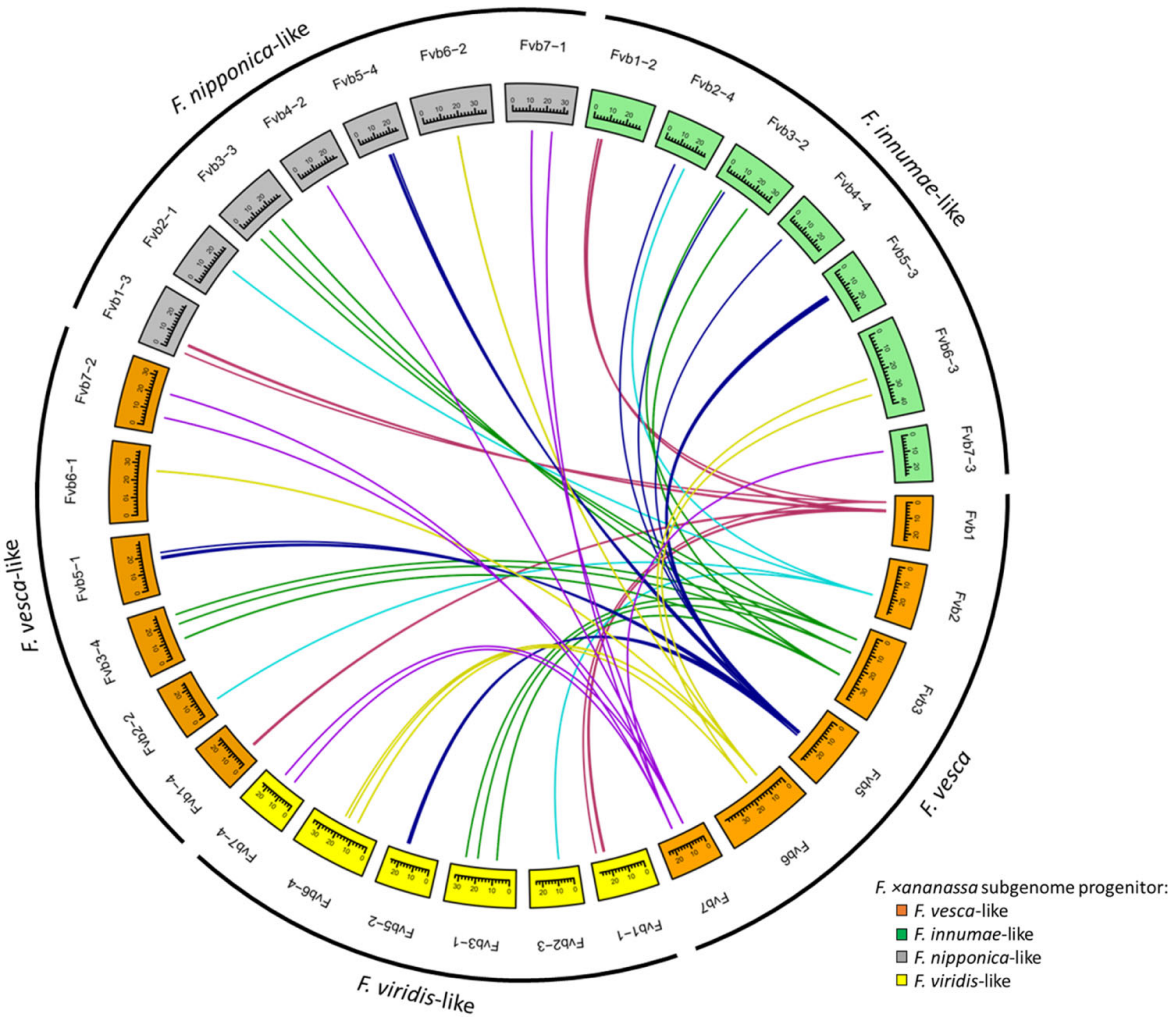
Synteny analysis of *MLO* genes between *F. vesca* and *F. ×ananassa*. Syntenic regions present in each chromosome of *F. vesca* were filled with red, light blue, green, dark blue, yellow, and purple sequentially. A total of 68 connecting lines between two genomes denote syntenic chromosomal regions. The *F. vesca* chromosomes are highlighted in orange while *F. ×ananassa* subgenomes were highlighted according to their diploid progenitors namely *F. viridis* (yellow), *F. vesca* (orange), *F. nipponica* (gray) and *F. iinumae* (green). Chromosomes Fvb4 from *F. vesca*, and Fvb4–1 and Fvb4–3 from *F. ×ananassa*, were not included since no putative *MLO* genes were identified in those regions. The relative chromosome size was indicated by the unit, Mbp. Circular visualization of syntenic regions between *F. vesca* and *F. ×ananassa* was constructed using an R-package “Circlize”.

The four subgenomes of the modern cultivated strawberry are a result of allopolyploidization with four specific diploid progenitor genomes, which have subsequently undergone substantial subgenome conversion^39^. To elaborate on this recent discovery, we display synteny networks for putative MLO genes between diploid (*F. vesca*) and octoploid (*F. ×ananassa*) strawberry (Fig. 3). Most putative *FaMLO* orthologs co-localized to the same chromosome of the *F. vesca* genome. Additional *FaMLO* orthologs of *FveMLO12* and *FveMLO19* were identified in subgenomes Fvb4–4 and Fvb4–2, respectively. Because of the octoploidy of the cultivated strawberry, four homeologous genes are expected for each *FveMLO* gene. This was filled for 11 out of the 20 *FveMLO* genes: *FaMLO2*, *FaMLO3*, *FaMLO5*, *FaMLO10*, *FaMLO11*, *FaMLO13*, *FaMLO14*, *FaMLO15*, *FaMLO16*, *FaMLO17, and FaMLO20*. For the other *FaMLO* gene, variants in copy number of homeologous genes for each *FaMLO* were identified with two homeologs each for *FaMLO18*, three for *FaMLO1*, *FaMLO9*, and *FaMLO19*, five for *FaMLO4*, and six for *FaMLO12* (Fig. 3, Table S1), which suggest rearrangement in the octoploid genome. Variation in MLO gene distributions illustrates the wide diversity in the genome composition of octoploid strawberry. Furthermore, there were at least two *FaMLO* homeologs with 80% or more sequence similarity with *FveMLO* orthologs (Fig. S2).

### Domain organization and structure characterization of octoploid strawberry *MLO* genes

**Fig. 4:**
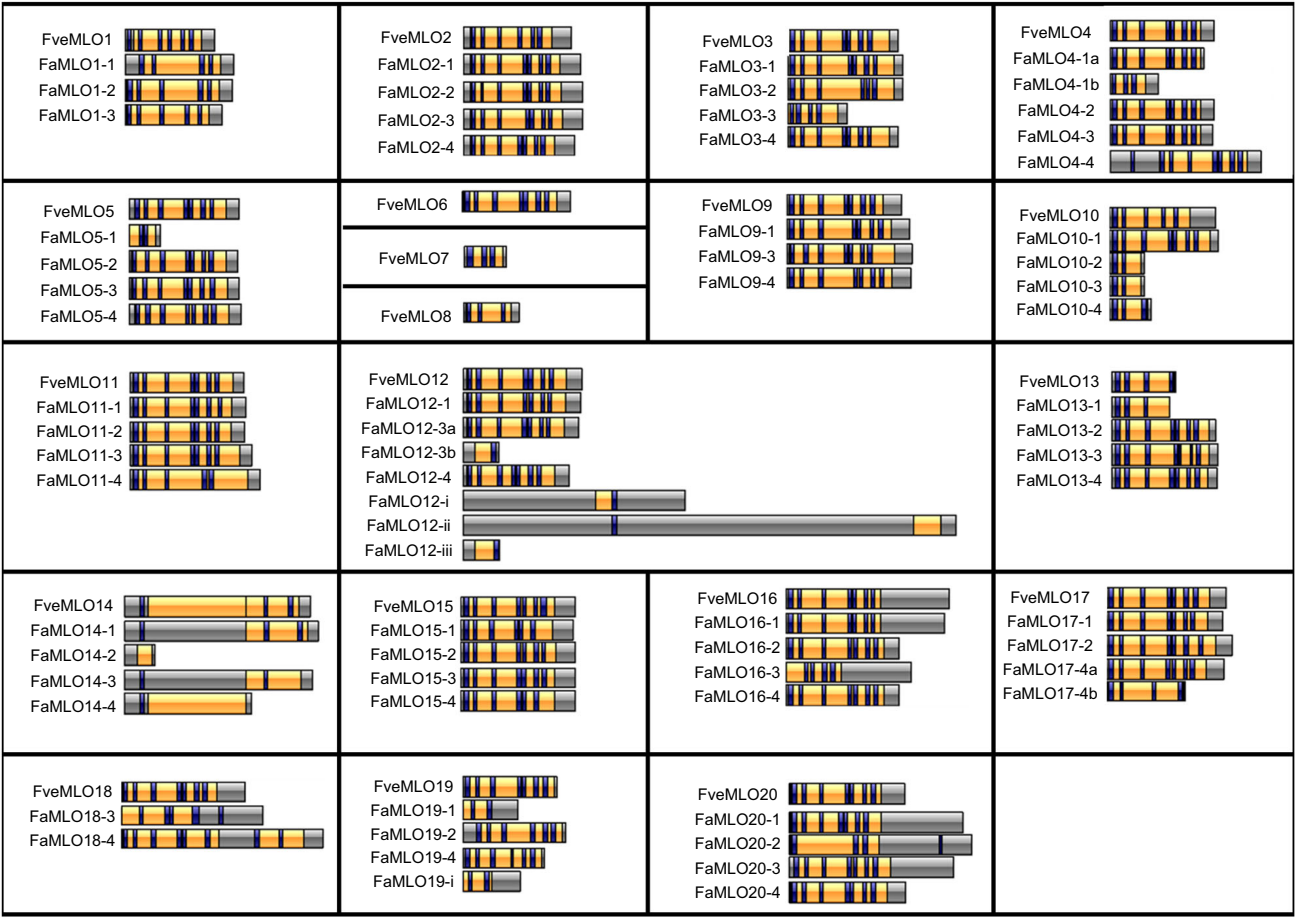
Domain organization of deduced MLO protein sequences of *F. vesca* and *F. ×ananassa*. Visualization of protein domains was constructed using IBS 1.0.3 (Illustrator for Biological Sequences) program^29^. The positions of conserved transmembrane (blue) and MLO (yellow) domains were predicted using online CCTOP prediction server (http://cctop.enzim.ttk.mta.hu/) and CDD: NCBI’s conserved domain database (https://www.ncbi.nlm.nih.gov /Structure/cdd).

The first *MLO* gene was identified in barley and was characterized as membrane protein with seven transmembrane (TM) domains and a uniquely-identified “*MLO*-functional domain”^1^. To examine the conserved protein domains of strawberry *MLO* genes, the deduced amino acid sequence of the predicted MLO proteins found in diploid and octoploid strawberries was subjected to theoretical domain prediction using online software InterProScan (https://www.ebi.ac.uk) and NCBI’s conserved domain database (https://www.ncbi.nlm.nih.gov /Structure/cdd). Most MLO proteins from either diploid or octoploid strawberry contain the conserved domain of MLO and TM, which covers a large portion of the protein (Fig. 4). To predict TM domains and subcellular localization of strawberry MLO proteins, CCTOP^44^ and WoLF PSORT^45^ software were used to find differences in the number of TM domains and subcellular localization among FveMLO and FaMLO proteins (Fig. 4, Table S2). All FveMLO proteins were predicted to localize within the plasma membrane, except for FveMLO7, which was predicted to localize in the extracellular matrix. Out of 68 *FaMLO* genes, 58 were predicted to localize within the plasma membrane, while nine were predicted to localize in other organelles: four in the chloroplast, two in ER, two in the nucleus, and one in Golgi bodies (Table S2). Thirteen out of 20 FveMLO proteins have seven TM domains, while seven have three to six TM domains. The *FaMLO* gene family has a high degree of variation in TM domain composition (Table S2). Only 35 FaMLO proteins have seven TM domains, while the remaining FaMLO proteins have TM domains ranging between zero and eight (Fig. 4, Table S2).

The domain organization of some FaMLO proteins showed high levels of conservation with the diploid ancestor species, *F. vesca*. For example, there were at least two homeologs of FaMLO2, FaMLO4, FaMLO5, FaMLO9, FaMLO11, FaMLO12, FaMLO15, FaMLO16, FaMLO17, and FaMLO20 that showed *F. vesca*-like domain structures (Fig. 4). However, one to three homeologs of some FaMLO proteins, including FaMLO3, FaMLO4, FaMLO5, FaMLO10, FaMLO12, FaMLO13, FaMLO14, FaMLO16, FaMLO17, FaMLO18, FaMLO19, and FaMLO20, showed more diverse protein structures indicating a distinct origin from different diploid progenitors (Fig. 4).

To further investigate the candidate susceptibility-conferring *FaMLO* genes, namely *FaMLO10-1*, *FaMLO10-2*, *FaMLO10-3*, *FaMLO10-4*, *FaMLO17-1*, *FaMLO17-2*, *FaMLO174a*, *FaMLO17-4b*, *FaMLO20-1*, *FaMLO20-2*, *FaMLO20-3*, and *FaMLO20-4*, their deduced amino acid sequences were aligned with their orthologs from *F. vesca* and *AtMLO2, AtMLO6*, and *AtMLO12* from Arabidopsis (Fig. S3-A). We identified conserved domains, including seven TM, CaMB and two C-terminal protein (I and II) domains of *MLO* genes^2,46^. FaMLO17-2, FaMLO17-4a, FaMLO20-1, FaMLO20-2, FaMLO20-3, and FaMLO20-4 proteins possessed seven TM domains and conserved CaMB and C-terminal I and II domains; however, FaMLO10-1, FaMLO10-2, FaMLO10-3, FaMLO10-4, FaMLO17-1, and FaMLO-17a- and FaMLO17-4b showed variable truncated protein sequences at the C-terminal end, resulting in the loss of these domains (Fig. S3-A). Among candidate *MLO* proteins, FaMLO10-1, FaMLO17-1, FaMLO17-2, FaMLO17-4a, FaMLO20-1, FaMLO20-2, FaMLO20-3, and FaMLO20-4 protein sequences are most identical to known susceptibility-conferring Arabidopsis MLO proteins, and therefore could have potential association with PM resistance in octoploid strawberry. MLO susceptibility factors were functionally conserved between monocot and dicots^47^. Among FaMLO12 proteins that were clustered in clade IV, FaMLO12-1, FaMLO12-3a and FaMLO12-4 showed high protein sequence identity and with functional MLO genes in distant relative monocot species (Fig. S3-B).

### Expression profiling of *FaMLO* genes in different tissues of cultivated strawberry

**Fig. 5:**
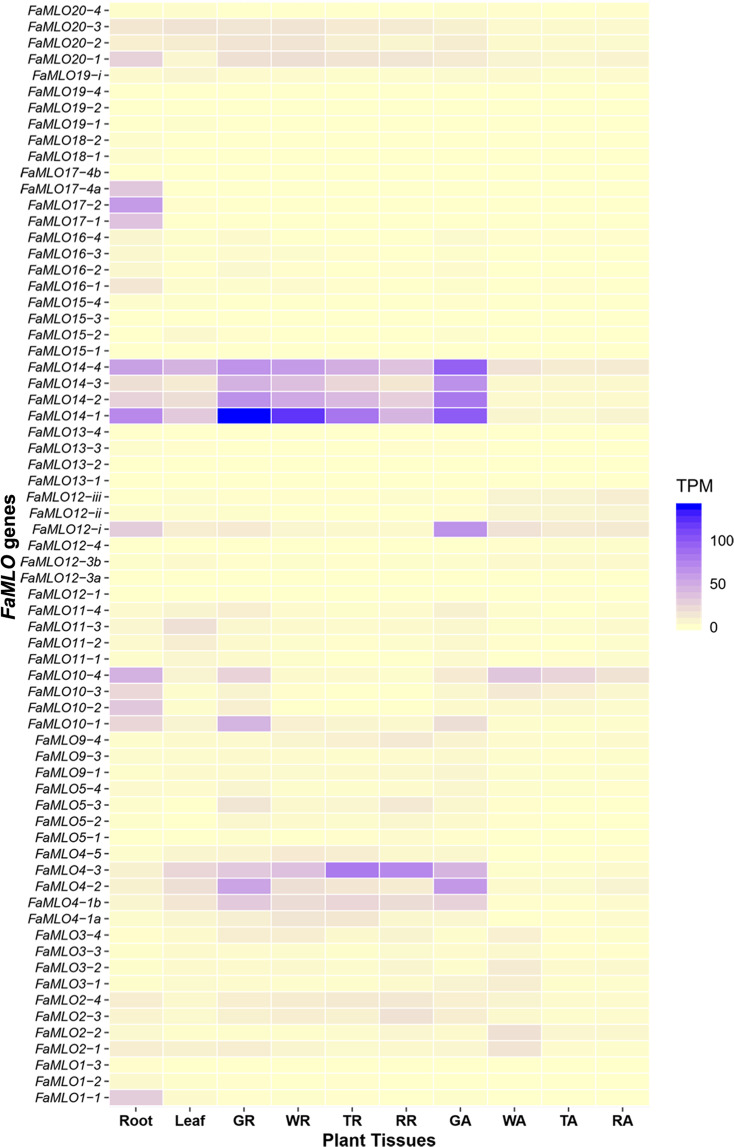
Expression profile of putative strawberry MLO genes in different plant tissues, e.g., leaf, roots, green (GR), turning (TR), white (WR), and red (RR) receptacles, and green (GA), turning (TA), white (WA), and red (RA) achenes extracted from RNA-seq data published by Sánchez-Sevilla, et al. (2017). The mean gene expression level was normalized using transcripts per million (TPM). Gene expression profile was visualized with heatmap using R-package “ggplot2”.

Expression patterns of previously characterized *MLO* genes suggested diverse biological functions for the *MLO* gene family^31^. To characterize *MLO* transcript accumulation patterns in octoploid strawberry, raw data from the strawberry gene expression atlas study^32^ were reassembled using the recently published octoploid strawberry genome^39^. The steady state accumulation of several *MLO* transcripts is specific to different tissues, with *FaMLO* genes such as *FaMLO1* and *FaMLO17* showing root-specific gene expression (Fig. 5, Table S4). Variation in gene expression between homeologs is also observed. For example, *FaMLO1–1* is highly expressed specifically in root, but the other homeologs, *FaMLO1–2* and *FaMLO1–3*, are not expressed. For candidate *FaMLO*s that are potentially associated with PM disease, the expression pattern and levels of transcript abundance also vary. The four homeologs of *FaMLO10* are expressed in roots at different levels while only *FaMLO10-1* and *FaMLO10-4* are preferably expressed in leaf and other developing fruit tissues. In root, *FaMLO17-2* also shows higher gene expression as compared with its other three homeologs, *FaMLO17-1* and *FaMLO174a*. The expression of *FaMLO17-4b* was not detected. The homeologs *FaMLO20-1, FaMLO20-2, and FaMLO20-3* show stable expression in root, leaf, and developing fruit tissues; however, *FaMLO20-4* is not expressed in any of these tissues (Fig. 5, Table S4).

### Structural variation analysis of putative *FaMLO* genes using long-read sequencing

Genetic mutation that causes allelic variation is a major driver of genetic diversity and change in gene function. To investigate gene diversity among putative *FaMLO* gene members of clade V, we determined genomic sequences of *FaMLO10*, *FaMLO17*, and *FaMLO20*, including their respective homeologs using long-read sequencing from one resistant University of Florida (UF) strawberry advance breeding selection, ‘13.55-195’ and compared them to the genomic reference sequences of the susceptible cultivar, ‘Camarosa’. A total of 455,111 PacBio long reads (6,735,339,589 bp) were generated representing about 15× coverage of the *F. ×ananassa* genome (805 Mb) and the sequence-length distribution was summarized in Figure S4 with an average length of 17,642 bp.

**Fig. 6:**
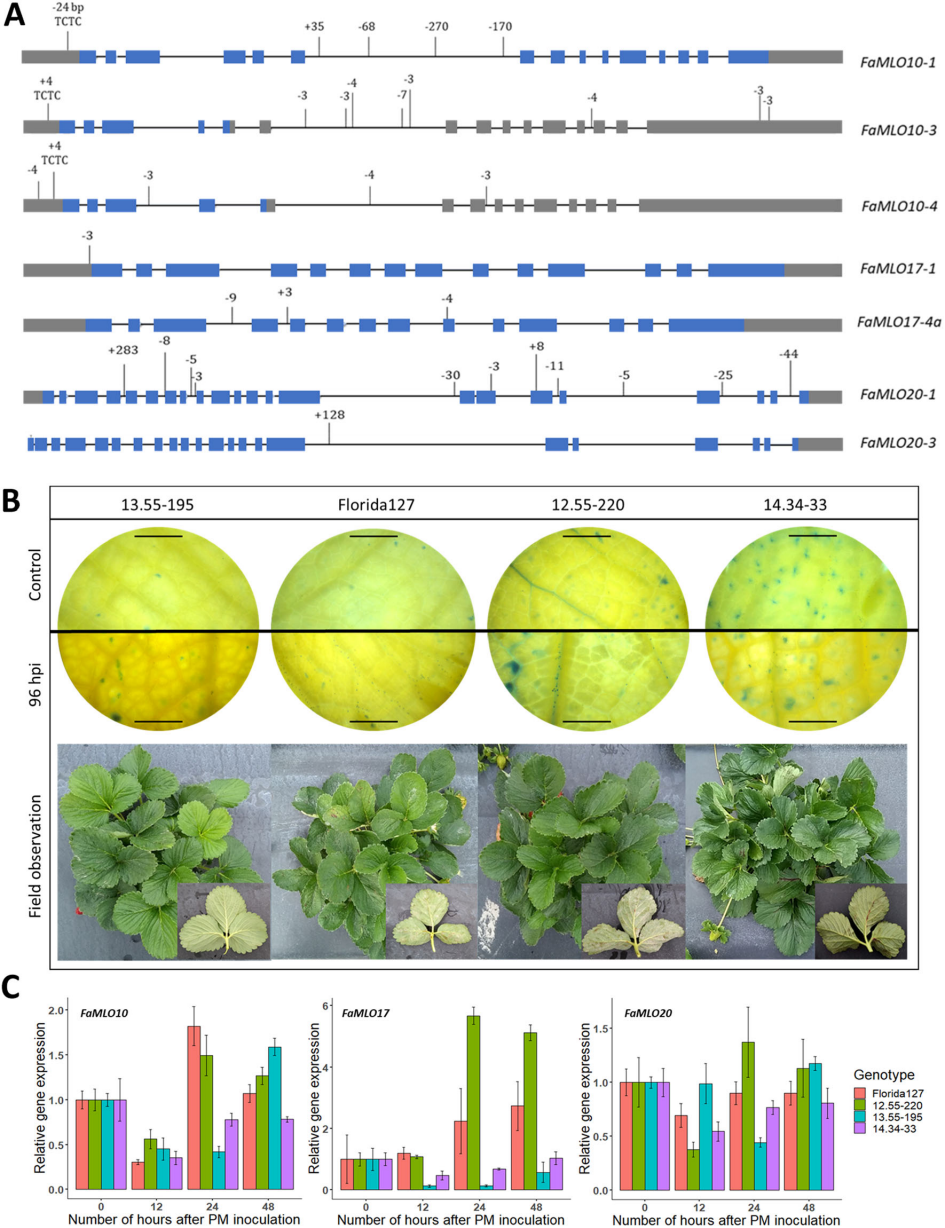
*FaMLO* associated with powdery mildew susceptibility in strawberry. **A** Structural variation of *FaMLO10*, *FaMLO17* and *FaMLO20* genes between ‘Camarosa’ and UF accession, ‘13.55-195’. The gene structure of *FaMLO* genes is shown with exons in blue boxes, UTR in gray boxes, and introns with a black line. The nucleotide insertions and deletions are presented by vertical lines with the corresponding number of indels. **B** Light micrographs of UF accessions, ‘13.55-195’, ‘Florida127’, ‘12.55-220’, and ‘14.34-33’ visualizing fungal infection at 0 (control) and 96 hpi (top). Leaf tissues were cleared in destaining solution and subsequently, the leaf segments were mounted on the slides and a drop of lactophenol cotton blue was added. The fungal structure can be visualized with cotton blue that specifically stains chitin in the fungal cell walls. Phenotype of four UF accessions in field conditions showing variable susceptibility/resistance to PM (bottom). **C** Relative gene expression of *FaMLO10* (top), *FaMLO17* (middle) and *FaMLO20* (bottom) in five UF accessions at 0, 12, 24, and 48 hpi by real-time qPCR. *FaGAPDH2* was used as internal control and plants at 0 hpi were used as sample control. Error bars indicate standard error.

The full length of 10 *FaMLO*-like sequences out of 12 candidate *FaMLO* genes was successfully extracted from the assembly of long read sequencing data from the resistant accession ‘13.55-195’ (Fig. S5). These include *FaMLO10-1*, *FaMLO10-3*, *FaMLO10-4, FaMLO17-1, FaMLO17-2, FaMLO17-4a*, and *FaMLO20-1* to *4*. The *FaMLO*-like genes were then used for pairwise comparison of genomic sequences with the reference genome ‘Camarosa’ and presented major sequence variations (SV) in seven *FaMLO-like* genes of resistant (‘13.55195’) accession (Fig. 6A). In *FaMLO10*, a wide SV with up to 269 bp was detected at the noncoding regions including a 24 bp deletion of TC repeats in 5′ UTR region of homeolog *FaMLO10-1*. Fewer SVs were detected in *FaMLO17* genes with indels ranging from 3 bp to 9 bp, while *FaMLO20* had a wider SV that reached up to 283 bp and homeolog *FaMLO20-1* having the most SVs. Furthermore, premature termination codon was detected from SV in *FaMLO17-4a* and *FaMLO20-1* (Table S5). Overall, these data provided evidence of high genetic differentiation of putative functional *FaMLO* genes that might be associated with adaptive response to PM.

### Expression analysis of *FaMLO* genes after *Podosphaera aphanis* inoculation

To evaluate functional putative *FaMLO* gene members of clade V, the level of transcript accumulation was determined using quantitative real-time PCR in leaf tissues of four strawberry accessions, including two susceptible, Sweet Sensation^®^ ‘Florida127’ and ‘12.55-220’, and two resistant accessions, ‘13.55-195’ and ‘14.33-34’, after PM infection. Three biological replicates were used for each following a randomized complete block design. Plants were inoculated with PM spores from naturally infected plants using a fine artist brush (Figure S6-B). Disease progression was observed among genotypes after PM infection and susceptible genotypes, ‘12.55-220’ and Sweet Sensation^®^ ‘Florida127’ showed rapid growth of conidia as compared with resistant genotypes (Fig. 6B, Fig. S6-C, D). Leaf tissues collected at 0, 12, 24, and 48 h post inoculation (hpi) were used for gene expression analysis.

The data result illustrated a variable transcript accumulation at different time points post inoculation among *FaMLO10*, *FaMLO17*, and *FaMLO20* genes (Fig. 6). *FaMLO10* was induced at 24 hpi in both susceptible genotypes, while the transcript level in resistant genotypes remained low (Fig. 6C). Surprisingly, *FaMLO17* that indicates a root-specific endogenous gene expression from RNA-seq analysis was highly upregulated in one susceptible genotype from 24 hpi to 48 hpi, while the resistant genotypes showed a low level of transcripts across multiple time points (Fig. 6C). A slight upregulation of the transcript was observed in one susceptible genotype, ‘12.55-220’ at 24 hpi but the rest did not show any obvious differences in *FaMLO20* gene expression observed between susceptible and resistant genotypes after PM infection (Fig. 6C). The primer pairs used in this experiment were not homeologous-specific, and use of homeologous-specific primer pairs could facilitate identification of specific homeologs of functional *FaMLO* genes (Table S6). Overall, the gene expression analysis in this study provided insights of potential *FaMLO* genes associated with susceptibility response to PM infection.

### Transient gene silencing of *FaMLO10* and *FaMLO20* genes in strawberry

**Fig. 7:**
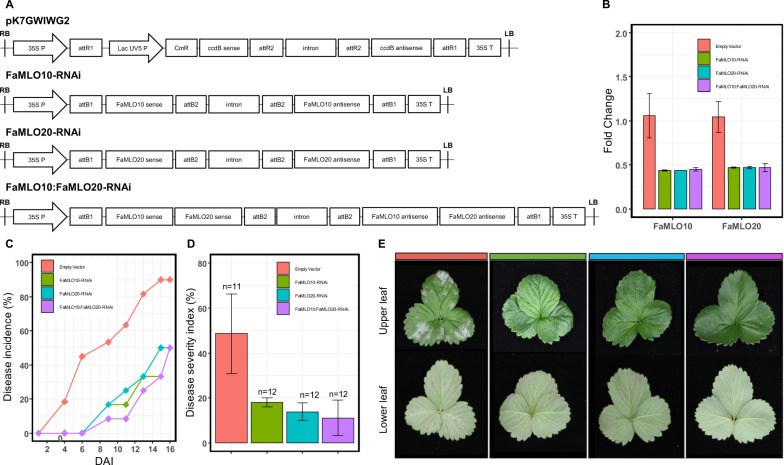
Transient gene expression silencing of *FaMLO10* and *FaMLO20* genes. **A** Construction of RNAi-expression vectors for transcriptional gene silencing in *F. ×ananassa* using UF accession ‘Sensation^®^Florida127’. **B** mRNA levels for *FaMLO10* and *FaMLO20* in silenced and nonsilenced strawberry. *FaGAPDH2* was used as internal gene control for −∆∆Ct normalization and gene expression level was calculated using 2. Bars represent mean ± standard error (SE) of three biological replicates with three technical replicates. **C** Progression of PM from 1 to 16 days after inoculation (DAI). **D** Evaluation of disease severity of leaf samples based on mycelial leaf coverage, and **E** leaf phenotypes of silenced and nonsilenced strawberry. Photos were taken at 16 DAI.

The constitutive expression of *FaMLO10* and *FaMLO20* in the leaf and the upregulation of these genes after pathogen inoculation showed potential effects in PM response. To further validate the functional role of these genes in PM susceptibility, we designed three RNAi constructs targeting *FaMLO10*, *FaMLO20*, and both *FaMLO10* and *FaMLO20* genes and transiently expressed each gene into a susceptible UF strawberry accession, ‘Sensation^®^Florida127’. The RNAi vector construction is summarized in Fig. 7A. A total of 11–12 seedlings were transformed for each construct, including the empty vector that was used as the nonsilenced control. The mRNA level reduced by more than 50% in silenced individuals compared with the control (Fig. 7B). However, the individual RNAi construct was sufficient to silence the other MLO gene, indicating a relatively high coding DNA sequence similarity between *FaMLO10* and *FaMLO20*. The transformed individuals were then inoculated with powdery mildew that was previously discussed and each transformant was evaluated for PM symptoms. The onset of PM symptoms of the nonsilenced control showed as early as five days after inoculation (DAI) and the symptoms rapidly progressed up to 90% at 16 DAI. On the other hand, the silenced individuals substantially delayed the progression of PM symptoms with only 50% disease incidence at 16 DAI (Fig. 7C). The nonsilenced control also showed higher DSI of more than 50% as compared with the silenced genotypes with DSI between 10% and 15%. (Fig. 7D, E). This finding suggests a major role of FaMLO10 and FaMLO20 genes in strawberry susceptibility against PM pathogen.

## Discussion

The *MLO* gene family is an important target in many agricultural crops for the improvement of resistance against PM pathogens. Previous characterizations identified that some *MLO* genes are involved in PM susceptibility, and that loss-of-function of those genes can confer durable and broad-spectrum resistance. In this study, it was found that variants of *F. vesca MLO* genes and *F. ×ananassa MLO* genes were distributed across the genome. However, we observed copy number variation of *FaMLO* homeologs showing differences in composition among subgenomes, supporting the distinct origin of each subgenome during the evolution of octoploid strawberry^29^. The unique *FaMLO* sequences in chromosome four (Fig. 1B and Fig. 3) that are not present in *F. vesca* might suggest an acquisition of chromosome segments from other diploid progenitors. Also, we did not find any significant homologs for *FveMLO6, FveMLO7*, and *FveMLO8* using the *F. ×ananassa* ‘Camarosa’ reference genome (Fig. 1B). An over representation of some *F. vesca*-like *MLO* gene homeologs in octoploid strawberry was detected and this coincides with the previous findings showing an evidence of the dominance of *F. vesca* subgenome during homeologous exchange^39^. The loss and gain of genes could be evolutionarily significant in the development of polyploid species^48^ or other possible functional divergence during domestication. Additional octoploid reference genomes would explain such phenomena, as the chromosomal localization of *FaMLO* genes and possible misorientation in genome sequence for some subgenomes can be identified.

Phylogenetic analysis grouped strawberry *MLO* genes into eight different clades (Fig. 2B), which is consistent with previously classified *MLO* gene families in diploid strawberry^41^ and other Rosaceous crops^5^. The phylogenetic analysis of *MLO* genes between octoploid and diploid strawberry spp. illustrated the genetic diversity among *FaMLO* genes, and often indicated an *F. vesca-*like sequence ancestry for most *FaMLO* genes due to subgenome conversion (Fig. 2A). *FveMLO* genes were closely related to *FaMLO* genes localized in *F. vesca*-like subgenomes (Fig. 2B), while *FaMLO* gene localized in either *F. iinumae*- and *F. nipponica*-like and *F. viridis*-like subgenomes that appeared to have greater diversity of *MLO* genes in terms of potential gene duplication (Figs. 2B and 3). Unique homeologs of *FaMLO4*, *FaMLO12, FaMLO17*, and *FaMLO19* were identified in these subgenomes supporting a greater genomic diversity of the genomes derived from these diploid progenitors (*F. iinumae* and *F. viridis*)^49^ that might explain the additional *MLO* copies in these subgenomes. Since genomes of homoploid hybrid lineages of *F. ×ananassa* were colinear and diploidized^49^, the genetic variation identified between *FaMLO* homeologs could be mainly attributed to their ancestral diploid progenitors and not by allelic diversity during chromosomal exchange between subgenomes.

Functional *MLO* genes associated with PM resistance are exclusively grouped in clades IV and V for monocots and dicots, respectively^7^. Interestingly, we found *FveMLO12*, six homeologs of *FaMLO12* grouped together in clade IV with previously characterized *HvMLO, ZmMLO1*, and *PpMLO12*^5^. In the present study, we found at least three clade V *FveMLO* and *FaMLO* genes, which are potential candidate genes involved in PM resistance. *MLO*-based resistance to the PM pathogen *P. aphanis* in a strawberry was successfully tested through RNAi-induced gene silencing of *FaMLO* genes using antisense *MLO* from peach (*PpMLO1*)^50^. In Arabidopsis, mutation of *AtMLO2* showed only partial resistance, while complete resistance was obtained after knocking out both *AtMLO6* and *AtMLO12*^13^.

The average MLO protein length (~500 amino acids) was similar in both *F. vesca* and *F. ×ananassa*, although few *FaMLO* proteins had more structural variations (SV) as compared with other homologous genes (Fig. 4). SV is defined as a change in genome sequence such as deletion, insertion, and inversion caused by mutations which in turn can affect gene function^51^. Furthermore, SV between homeologs of *FaMLO10* and *FaMLO12* causes changes in their gene expression profile (Fig. 5) and subcellular localization (Table S2). SV between homeologs might result in different gene regulation of gene expression and consequently lead to novel functions^43^. Diverse gene expression patterns among *FaMLOs* across different tissues (Fig. 5) suggest that MLO proteins might be specialized for different biological responses. This may be analogous to the unique expression patterns observed for Arabidopsis *MLO* genes, where transcription of each *MLO* gene is distinct and regulated differently by various biotic and abiotic stimuli^52^. In the present study, we found that the number of the TM domains of *FveMLOs* and *FaMLOs* varied between zero and eight. This variation was also observed in previous genome-wide *MLO* studies in other Rosaceous crops such as apple and peach^5^. Redundancy of gene function among homologs could arise from gene duplication for adaptation to varying selection pressures and changing environments^53,54^. High gene copy number is commonly observed in polyploid crop species due to the presence of homeologous genes, posing a considerable challenge for determining the biological contribution of specific genes. The genes *FaMLO10-1, FaMLO17-1, FaMLO17-2, FaMLO17-4a, FaMLO17-4b,* and *FaMLO20-1 to 4* showed the highest sequence identity with known Arabidopsis *MLO* genes. Like functional *AtMLO* genes, we detected a transcriptional upregulation of *FaMLO* genes upon challenge of strawberry powdery mildew (Fig. 6C). Silenced MLO genes in strawberry also showed substantial delay in disease progression with reduced PM symptoms (Fig. 7C–E), indicating potential role in PM susceptibility^16,55^. Furthermore, genomic sequence variations in these *FaMLO* genes between resistant and susceptible genotypes were detected, including a truncation in TC repeats in the promoter region of the gene (Fig. 6B) that is known be evolutionarily conserved among functional *MLO* genes^56^. Like in the previous studies, silenced MLO genes in strawberry showed substantial delay in disease progression with reduced PM severity (Fig. 7C–E)^16,55^. Meanwhile, three homeologs of *FaMLO12* genes that were closely related and shared conserved protein domains with the known *MLO* genes to confer to PM susceptibility in monocot were also identified as potential functional *MLO*-susceptible genes. Since, the susceptibly factors in *MLO* genes were functionally conserved between monocot and dicot^47^, this suggests that they might also contribute to strawberry MLO susceptibility. Overall, we identified a total of nine and three *FaMLO* genes with high homology to functional disease-susceptible *MLO* in dicot and monocot, respectively, representing candidate genes for breeding new cultivars with improved PM resistance. These putative *FaMLOs* should be further validated for their functional roles in PM resistance with more breeding accessions and cultivars.

## Conclusions

Here, we identified a total of 20 *MLO* homologs in *F. vesca* and 68 in *F.×ananassa*. Three *FveMLO* genes and ten *FaMLO* genes were clustered with previously characterized *MLO* genes known to be in PM resistance/susceptibility in other plant species. The deduced amino acid sequences of putative strawberry *MLO* genes showed conserved protein characteristics, including transmembrane and calmodulin-binding domains that have been previously described. The considerable amino acid-level variation between MLO homoeologous copies was observed, suggesting possible non-redundant functions of MLOs in different subgenomes. *FaMLO10, FaMLO17* and *FaMLO20* are the most identical to functionally characterized *MLO* genes associated with PM susceptibility. The expression of *FaMLO10* and *FaMLO17* was substantially induced in response to the infection of *P. aphanis* in susceptible strawberry varieties and RNAimediated silencing of FaMLO10 and FaMLO20 greatly delayed disease progression and reduced PM severity suggesting a potential functional role in the PM resistance. Moreover, sequence variations in these *MLO* genes were detected between resistant and susceptible cultivars, and can be a potential target for functional characterization via CRISPR gene editing in future studies. Taken together, these data are a critical first step in understanding the allele function of the strawberry *MLO* gene family and should be useful for future functional studies to better understand their role in powdery mildew resistance in strawberry.

## Materials and methods

### Identification of the strawberry *MLO* genes in diploid and octoploid strawberry

To identify *MLO* gene orthologs in diploid strawberry, *F. vesca*, Arabidopsis *MLO* genes were searched to the latest *F. vesca* v4.0.a1^40^ genome. Consequently, each *FveMLO* gene identified was used to search for predicted *MLO* genes in octoploid strawberry using the masked version of the *F. ×ananassa* v1.0.a1^39^ reference genome. The deduced diploid and octoploid strawberry protein sequences were validated by reciprocal BLAST searches obtained from NCBI data sets of Arabidopsis reference genome. The chromosomal localization of each predicted *MLO* genes in *F. vesca* and *F. ×ananassa* was identified using available information in GDR database and visualized using MapChart 2.3 software^57^. Gene structure featuring introns, exons and UTR of predicted *MLO* genes was constructed using Gene Structure Display Server 2.0 (GSDS) (http://gsds.cbi.pku.edu.cn/)^58^.

For phylogenetic analysis of *MLO* genes in *Fragaria* species, full-length *MLO*–like coding sequences were extracted from the *F. nipponica*^37^, *F. iinumae*^36^, *F. vesca*^40^, *F.viridis*^38^, and *F. ×ananassa*^39^ genomes. The CIPRES Science Gateway^59^ was utilized for full-length coding sequence alignment using MUSCLE v3.7^60^ and Maximum likelihood analysis using RAxML v8.2.10^61^. Tree construction was performed with 100 bootstrap replicates and rooted with Arabidopsis *MLO1* (NM_116494), and this process was replicated five times using different random number seeds. Trees were visualized with a 50% bootstrap threshold using CLC Genomic Workbench 11.

For phylogenetic analysis of strawberry *MLO* and functional *MLO* genes, the protein sequences of *MLO* genes from *F. vesca* and *F. ×ananassa* were aligned to other available sequences of plant species, and phylogenetic relationship was constructed using FastTree consensus tree protein alignment via Geneious^®^ 11.0.5 software^62^. Phylogenetic tree was constructed by adding 15 *MLO* genes from Arabidopsis, six *MLO* from other Rosaceous crops, apple (*MdMLO*s) and peach (*PpMLOs*), barley (*HvMLO*), corn (*ZmMLO1*), rice (*OsMLO1*), tomato (SlMLO1) and pepper (*CaMLO2*).

### *d*N/*d*S analysis of *MLO* genes in *Fragaria* species

Putative orthologous *MLO* genes from the *F. ×ananassa*^39^, *F. vesca* v4^40^, *F. iinumae*^36^, and *F. nipponica*^37^ genomes were identified from the Maximum Likelihood phylogenetic tree analysis. Alignment of each orthologous protein pair was performed using MUSCLE v3.7^59^ followed by PAL2NAL (v14)^63^ supplied with the corresponding DNA sequences to convert the peptide alignment to a codon alignment. The *d*N and *d*S values were computed using the codeml program from PAML^64^ implemented on the PAL2NAL web server (http://www.bork.embl.de/pal2nal).

### Synteny analysis of strawberry *MLO* genes

Pairwise comparisons of coding DNA sequence (CDS) between predicted *FveMLO* and *FaMLO* genes were obtained using ClustalW2 multiple-sequence alignment via Geneious software^62^ followed by heat map visualization to determine closely related *MLO* genes using Rpackage “Lattice”^65^. Synteny analysis of *MLO* genes between *F. vesca* and *F. ×ananassa* was summarized using R-package “Circlize”^66^.

### Protein characterization and domain prediction

The deduced amino acid sequences of putative *FveMLO* and *FaMLO* genes were analyzed by different prediction software to identify functional domains and determine protein topologies and sub-cellular localizations. Functional MLO domains of protein sequences were predicted using CDD: NCBI’s conserved domain database (https://www.ncbi.nlm.nih.gov /Structure/cdd)^67^. Protein topology and number of transmembrane domains were predicted using online software CCTOP Prediction Server^44^ while protein sub-cellular localization was analyzed using WoLF PSORT program^45^. Default setting was used to run for all prediction software. Visualization of protein domains was constructed using IBS 1.0.3 (Illustrator for Biological Sequences) program^68^. To analyze conserved amino acids of *MLO* genes associated with PM resistance, protein sequences of *FveMLO10*, *FveMLO17*, and *FveMLO20* from *F. vesca* and *FaMLO10*, *FaMLO17*, and *FaMLO20* from *F. ×ananassa* were aligned against functionally characterized *AtMLO2*, *AtMLO6* and *AtMLO12* from Arabidopsis using MultAlin software (http://multalin.toulouse.inra.fr/multalin/)^69^.

### Expression profile of putative strawberry *MLO* genes

To examine transcript accumulation patterns of putative strawberry *MLO* genes, RNAseq libraries from various ‘Camarosa’ tissues^70^ with the study reference PRJEB12420 were downloaded from the European Nucleotide Archive (https://www.ebi.ac.uk/ena). The complete 54 libraries RNA-seq experiment consisted of six independent green receptacle libraries, six white receptacle libraries, six turning receptacle libraries, six red receptacle libraries, three root libraries, three leaf libraries, and six achene libraries each for all the corresponding fruit stages. For both libraries, raw RNA-seq reads were assembled to the ‘Camarosa’ reference genome using CLC Genomic Workbench 11 (mismatch cost of 2, insertion cost of 3, deletion cost of 3, length fraction of 0.8, similarity fraction of 0.8, and 1 maximum hit per read). Reads that mapped equally well to more than one locus were discarded from the analysis. RNA-seq counts were quantified in transcripts per million (TPM).

### Genomic DNA extraction and PacBio sequencing

Leaves of UF strawberry-breeding selection, ‘13.55-195’, were kept in the dark for a week and etiolated leaf tissues were collected for DNA extraction. The DNA was extracted using a modified CTAB method^71^ and sheared to about 20 Kb. The construction of SMRTbell libraries and sequencing a total of 14 SMRT cells using a PacBio RSII sequencer (Pacific Biosciences) were conducted at UC Davis Genome Center. After sequencing, RSII raw bam files were converted into subreads in FASTA format using the standard PacBio SMRT software package and produced a total of 11.97 Gb (~15x coverage of *F. ×ananassa* genome) with average read length of 19,957 bp.

### Genome assembly of long-read PacBio sequences

The 11.97-Gb subreads generated from PacBio sequencing were assembled using Canu assembler^72^ (corrected error rate = 0.105, cor out coverage = 200). A total of 12,174 contigs were generated with an N50 of 17.64 Kb (Fig. S4).

### *Podosphaera aphanis* inoculation using naturally infected strawberry leaves

In this study, a total of five UF strawberry accessions, including one cultivar (‘Sensation^®^Florida127’) and four advance breeding selections, ‘12.55-220’, ‘13.55-195’, ‘13.42-5’, and ‘14.33-34’, were used for *FaMLO* gene expression analysis. Of these, two genotypes, ‘Sensation^®^Florida127’ and ‘12.55-220’ were susceptible while the rest were resistant to PM. All accessions were transplanted in small pots in the greenhouse and were maintained pathogen-free for few weeks before fungal inoculation.

The inoculation using naturally infected strawberry leaves was performed following a modified method described by Calis, et al.^73^. The PM isolates were propagated and maintained on a susceptible strawberry genotype in a growth chamber (Fig. S6-A, B). Inoculation with PM was done using a fine artist’s paintbrush to carefully brush conidia from heavily infected leaves onto the leaves. Brushing was carried out at a height of ∼5–10 cm above the canopy to achieve an even distribution of conidia. The plots were arranged in an RCB design with three independent biological replicates and strawberry plants were well-watered and were maintained in a desirable condition with a temperature and humidity of ~70°F and 60–70%, respectively, throughout the experiments. The deposition of conidia was estimated by counting the number of spores in a 1-mm^2^ area under a microscope (Figure S6-C). Leaf tissues (S1 leaf) were collected at 0, 12, 24, 48, 72, and 96 h post inoculation (hpi) and immediately kept in a −80 °C freezer before processing.

### RNA extraction and expression analysis using Real-Time Quantitative PCR (qPCR)

The leaf tissues were ground using liquid nitrogen and the total RNA was extracted using Spectrum™ Plant Total RNA Kit (Sigma–Aldrich, MO, USA) as recommended by the manufacturer. To remove any DNA contaminants, the isolated RNA was treated with DNase I (Invitrogen, MA, USA). The RNA samples from 0, 12, 24 and 96 hpi were selected for expression analysis using Real-Time qPCR. A total of 1 µg of total RNA was used for firststrand cDNA using LunaScript^®^ RT SuperMix Kit (New England Biolabs, MA, USA). The target genes were *FaMLO10*, *FaMLO17*, and *FaMLO20*, while *FaGAPDH2* was selected as endogenous control. Primers were designed using IDT PrimerQuest tool (https://www.idtdna.com/PrimerQuest/Home/Index) and all sequences were presented in Table S5. The qRT-PCR experiment was performed using LightCycler^®^ 480 system (Roche, Swizerland) using Forget-Me-Not™ EvaGreen^®^ qPCR Master Mix (Biotium, CA, USA). The Real-Time qPCR reaction was performed in triplicates of 100 ng of cDNA, 0.4 µl of each primer (400 nmol), and 3 µl of EvaGreen master mix in a final volume of 5 µl. The primer sequences were presented in Table S5. The reaction conditions were 95 °C for 5 min, followed by 40 cycles of 95 °C for 20 s, 60 °C for 20 s and 72 °C for 20 s followed by melting curve analysis to validate the single-amplicon product. The relative gene expression was calculated by using the Livak method (2^−∆ΔCT^)^74^.

### RNAi vector construction and bacterial transformation

Using pssRNAit^75^, two ~200-bp RNAi fragments of genes *FaMLO10* and *FaMLO20* and one combined ~400-bp fragment were synthesized (Eurofins, Des Moines, IA, USA) and cloned in pDONR221™ vector (Thermo Fisher Scientific, Waltham, MA, USA) using standard procedures (Table S7). The insert identity was confirmed by sequencing (GENEWIZ, South Plainfield, NJ, USA) of three clones for each target gene. After checking, the fragments were inserted into silencing RNAi Gateway^®^ vector pK7GWIWG2(II)^76^ and the transformants were confirmed by sequencing both strands. The constructs were then inserted into *Agrobacterium tumefaciens* strain EHA105 and the transformed cells were tested by PCR using specific primer pairs for the presence of RNAi constructs (Table S7).

### Agrobacterium-mediated leaf transformation

The method for transient leaf transformation was performed as described by Cui et al.^77^ with slight modifications. One-month-old cultivated octoploid strawberry seedlings, ‘Sensation^®^Florida127’ from the University of Florida were used for leaf transformation. The seedlings were submerged in a 400-ml glass beaker with 350-ml bacterial suspension and a vacuum was applied. The vacuum was kept at 1.3 × 10^−8^ MPa for 3 min and was slowly released to facilitate Agrobacterium infiltration in the leaf. The excess bacterial suspension on the leaf surfaces was removed using filter paper. The seedlings were placed in the growth chamber with ambient and normal conditions (22 °C with RH of ~70%, 14 h of light, and 10 h of dark daily cycle). The seedlings were kept for five days in the growth room, and then leaf samples were collected for RNA isolation.

### Transcript and phenotypic analysis of silenced FaMLO genes

As described previously, mRNA levels of genes FaMLO10 and FaMLO20 were quantified using qRT-PCR in silenced and nonsilenced strawberry. The remaining seedlings were then infected by PM pathogen, as previously described and were assessed for disease progression and PM severity symptoms up to 16 DAI. The disease severity was rated as described by Kennedy et al.^78^ using a modified Horsfall–Barratt scale of 0–6 based on mycelial leaf coverage with zero and six as the least (0%) and most severe (100%), respectively. The disease incidence (DI) and disease severity index (DSI)^79^ were calculated using the following formula:

$${\mathrm{DI}} = \left( {{\mathrm{number}}\,{\mathrm{of}}\,{\mathrm{infected}}\,{\mathrm{plants}}/{\mathrm{total}}\,{\mathrm{number}}\,{\mathrm{of}}\,{\mathrm{plants}}} \right)100$$

$${\mathrm{DSI}} = \left[ {{\mathrm{sum}}\,{\mathrm{of}}\,{\mathrm{all}}\,{\mathrm{disease}}\,{\mathrm{ratings}}/\left( {{\mathrm{total}}\,{\mathrm{number}}\,{\mathrm{of}}\,{\mathrm{ratings}} \times {\mathrm{maximum}}\,{\mathrm{rating}}} \right)} \right] \times 100$$

## Supplementary information

Table S1

Table S2

Table S3

Table S4

Table S5

Table S6

Table S7
